# The evaluation of Rolimeter, KLT, KiRA and KT-1000 arthrometer in healthy individuals shows acceptable intra-rater but poor inter-rater reliability in the measurement of anterior tibial knee translation

**DOI:** 10.1007/s00167-021-06540-9

**Published:** 2021-03-31

**Authors:** Armin Runer, Tommaso Roberti di Sarsina, Vasco Starke, Alessandra Iltchev, Gernot Felmet, Sepp Braun, Christian Fink, Robert Csapo

**Affiliations:** 1 Department of Orthopaedics and Traumatology, Medical University of Innsbruck, Innsbruck, Austria; 2 Clinica Ortopedica e Traumatologica, Istituto Ortopedico Rizzoli, Bologna, Italy; 3 Medical University of Innsbruck, Innsbruck, Austria; 4 Orthopädische Praxis and ARTICO Sportklinik, Schwenningen, Germany; 5 Gelenkpunkt - Sports and Joint Surgery, Innsbruck, Austria; 6 Medical Informatics and Technology, ISAG, Research Unit for Orthopaedic Sports Medicine and Injury Prevention, Private University for Health Sciences, Innsbruck, Austria

**Keywords:** Knee arthrometer, Anterior cruciate ligament, *ACL* anterior tibial translation, Side-to-side difference, Equivalence testing; Lachman Test, Rolimeter, KiRA, KLT, KT1000

## Abstract

**Purpose:**

To assess measurement equivalence, inter- and intra-rater reliability, standard error of measurements (SEM) and false positive measurements (FPM) of four different knee arthrometers (KLT,Karl Storz; KiRA, I + ; KT-1000 MEDmetric Corp; Rolimeter, Aircast) in healthy patients.

**Methods:**

Four different investigators (two advanced (AR) and two beginners (BR)) examined 12 participants with healthy knees at two time points with regards to anterior tibial translation (ATT) and side-to-side difference (SSD). Test equivalence was assessed using the TOST (two-one-sided *t* test) procedure with ± 1 mm equivalence boundaries. Intraclass correlation coefficients (ICCs) were calculated using two-way mixed effects models. Furthermore, false positive-(SSD > 3 mm) and SEMs were assessed.

**Results:**

A total of 2304 Lachman Tests were performed. Between-rater SSDs were equivalent between AR and BR raters for the Rolimeter only. Inter-rater ICC values (SSD, ATT) were graded as “poor” to “moderate” for all devices. Equivalent test–retest results were observed for all raters using the Rolimeter, KLT and KT-1000, whereas measurement consistency with KiRA was given in the advanced examiners group only. Intra-rater ICC values (Range: SSD, ATT) were graded as “poor” to “moderate” for SSD values and “moderate” to “good” for ATT. SEMs were lowest for the Rolimeter and highest for KiRA. FPM were never obtained with the Rolimeter (0%), twice (2.1%) with the KT-1000, three times (3.1%) with the KLT and 33 times (34.4%) using KiRA.

**Conclusion:**

There is acceptable intra-rater but poor inter-rater reliability with all tested arthrometers. Measures of knee laxity are comparable between Rolimeter, KLT and KT-1000 but higher for KiRA. Clinically, the present study shows that repeated arthrometry measurements should always be performed by the same investigators.

**Supplementary Information:**

The online version contains supplementary material available at 10.1007/s00167-021-06540-9.

## Introduction

Instrumented measurements using arthrometers have become increasingly popular for objective assessments of knee laxity, and are widely used for pre–and postoperative evaluation of acute and chronic anterior knee instability [5, 9]. Arthometers can be used by both orthopedic surgeons and rehabilitation specialists during routine diagnostic clinical examination, to evaluate the effectiveness of treatment and especially in the field of research to facilitate comparisons of postoperative outcomes, as they help objectify the evaluation of knee laxity [5, 9]. Today, a broad variety of knee arthrometers is available, with the KT-1000 (MEDmetric Corp, San Diego, Calif., USA) and the KT-2000 (an updated KT-1000 with an X–Y-plotter) being the most widely used and studied devices. It has been shown to provide accurate and reproducible knee laxity measurements with inter- and intra-rater reliability ranging from 0.41 to 0.92 [4, 8, 10, 17, 18, 20], and 0.83 to 0.97, respectively [1, 5, 8–11, 17, 18, 21]. Similarly, the Rolimeter (Aircast Europa, Neubeuern, Germany) is an easy to use, simple and compact arthrometer that yields comparable knee laxity measurements as the KT-1000 [3, 9]. Unfortunately, both instruments are no longer commercially available. Therefore, new devices like the KLT (Karl Storz, Tuttlingen, Germany) or KiRA (I + , Italy) were introduced [19]. While one study comparing the measurement results of KiRA to those of KT-1000 showed comparable side-to-side differences (SSD) in anterior tibial translation (ATT), no studies published to date have evaluated and compared the results of KLT to other, frequently used arthrometers [19].

**Fig. 1:**
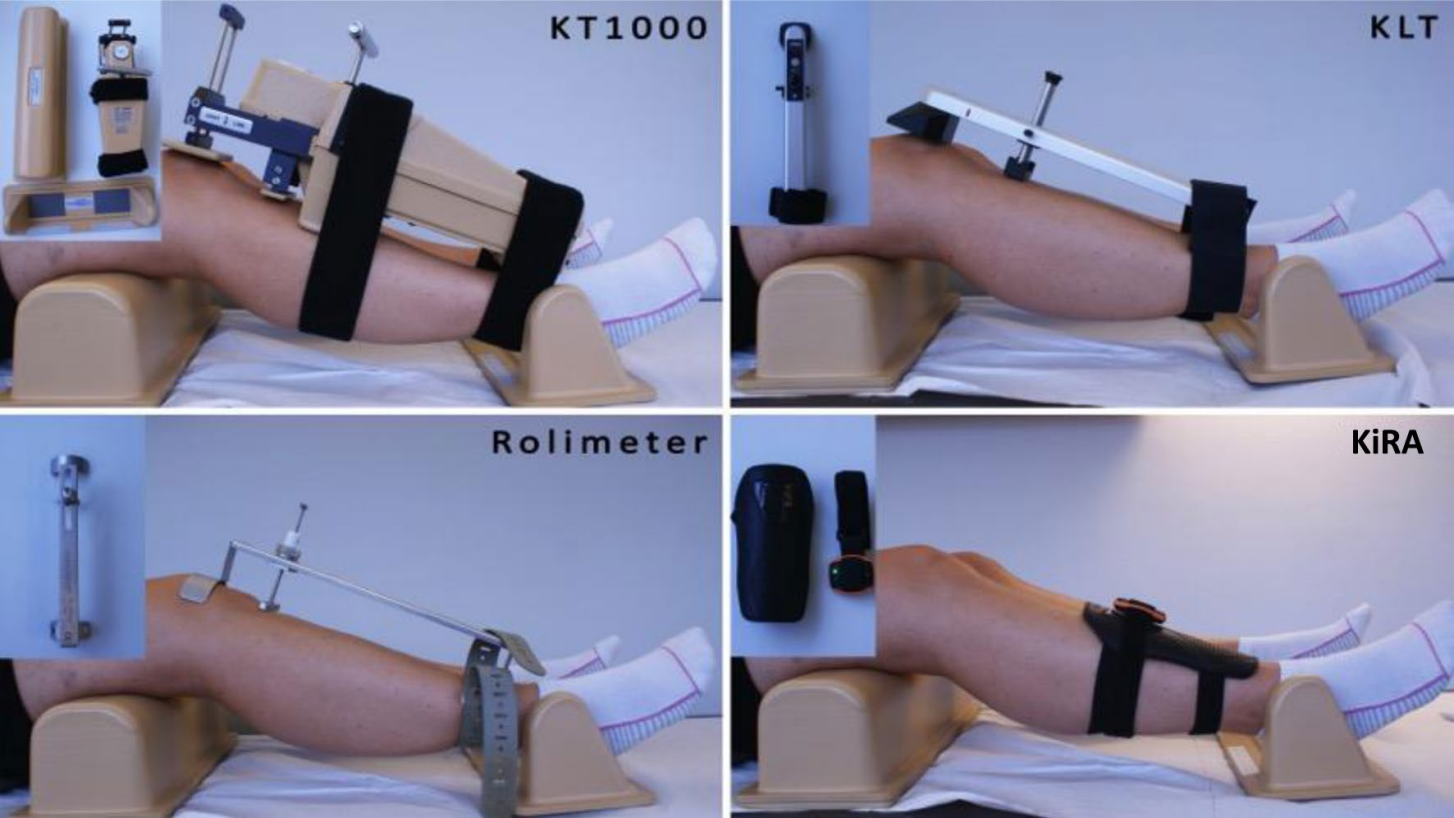
Test setup and patient positioning with all four arthrometers tested: KT-1000 (MEDmetric Corp, San Diego, Calif., USA), Rolimeter (Aircast, Europe), KLT (Karl Storz, Tuttlingen, Germany) and KiRA (I + Italy)

All above-mentioned devices (Fig. 1) provide linear measurements of anterior tibial translation (ATT); however, marked differences in handling- and test setups exist. It is well known that many factors including examiner experience, the kind of arthrometer used, device positioning and overtightening, force application, leg external/internal rotation, examiner hand dominance, pain, effusion as well as muscular guarding (e.g., through hamstring contraction) influence the measurement outcomes. To allow a meaningful comparison of results between different devices and measurement outcomes of different raters, knowledge about the arthrometers’ reliability is crucial.

The aims of this study were (i) to assess the equivalence of measurements, the intra- and inter-rater reliability as well as the device-specific standard errors of measurements (SEM) of four different arthrometers in healthy knees, and (ii) to compare the results of these arthrometers in four different raters with different experience levels (advanced raters vs. beginners) in terms of anterior tibial translation (ATT) and side-to-side differences (SSD).

## Materials and methods

The study was approved by the ethical committee of the Medical University of Innsbruck (EK Nr: 1256/2020) and conducted according to the Declaration of Helsinki. All participants agreed and signed the informed consent.

### Arthrometers

In the present study, the Rolimeter (Aircast Europa, Neubeuern, Germany), KLT (Karl Storz, Tuttlingen, Germany), KiRA (I + , Italy) and KT-1000 (MEDmetric Corp, San Diego, Calif., USA) arthrometers were used for testing.

Testing setups for the KT-1000 and Rolimeter have been described in detail elsewhere [6, 9]. Similar to the Rolimeter, the KLT is fixed to the lower leg with an adjustable ankle strap that is placed distally on the patient’s leg. While the proximal curved plate of the Rolimeter is placed on the patella and an adjustable stylus is positioned at the center of the tibial tuberosity, the KLT is aligned with the patient’s tibiofemoral joint line. A red reference line on the side of the device serves to align the arthrometer with the joint line (Fig. 1: KLT).

KiRA, a triaxonal accelerometer for rotational and translational laxity evaluation, can be used to perform both an instrumented Lachman and Pivot Shift test [19]. For ATT measurements, the arthrometer relies on a standard Lachman test. The device is placed at the distal lower leg of the patient and fixed with an elastic strap in contact with a shin guard in order to optimize the stability of the sensor (Fig. 1: KiRA).

### Study participants

A total of twelve 12 (six6 women) healthy participants without any known previous or ongoing knee as well as soft tissue pathology’s were included. Prior to inclusion, the clinical history was assessed and an extensive physical examination was performed to ascertain that participants were free of ACL injury. Their mean age and BMI were 28.6 ± 6.6 years and 22.9 ± 2.1 kg/m^2^, respectively. All subjects were tested at two different time points (Test–Retest). All study participants gave their written and oral informed consent prior to study inclusion.

### Examiners

A total of four examiners performed the study protocol. Examiners 1 and 2 were experienced orthopedic surgeons (advanced), with more than 5 years of experience preforming manual knee examination, while examiners 3 and 4 were students (beginner) in their final year of medical school. Both advanced users were experienced in the use of one of the four arthrometers (Examiner 1: KLT, Examiner 2: KiRA). The beginners, by contrast, were familiar with the execution of the clinical Lachman Test but had no experience in the use of arthrometers. For this reason, prior to the study, all examiners were instructed in the proper use of all arthrometers according to the respective user manuals, and given ample opportunity to familiarize with the handling of the devices.

### Study protocol

All participants were positioned in a standardized manner in supine position, with the knee flexed at 30° and fixed in a leg holder in order to keep the knee in neutral position (Fig. 1). Then, the arthrometers were attached to the lower leg as per the manufacturers’ instructions. Participants were told to fully relax and hamstring contracture was manually checked prior to every test. By manually applying anterior force to the proximal calf, three consecutive measurements (= one test battery) were obtained in both knees with all four devices. Each examiner tested and retested each participant within one day. Arthrometers were removed from the leg after each test battery and participants were allowed to stand up between measurements. In order tTo minimize the risk of bias, examiners were not allowed to see or read the analog or digital displays of the arthrometers showing the extent of anterior tibial displacement. Furthermore, the sequence of the examiners as well as the sequence of the arthrometers used were randomized across all participants.

### Statistical analysis

All data were analyzed using R Statistics (version 3.6.1, https://www.R-project.org/) and displayed as means and standard deviations (SD). The level of statistical significance was set to 0.05. The assumption of normality of data was tested using the Kolmogorov–Smirnov test.

Measurements of one test battery (three consecutive tests on the same leg) were averaged to obtain the mean ATT. SSDs were calculated by subtracting the mean ATT measurements of the left leg from those obtained in the right leg. SSD’s greater than 3 mm were considerate false positive measurements [6, 17].

Within-group equivalence of the mean results obtained by examiner 1 and 2 (advanced raters) as well as examiner 3 and 4 (beginners), respectively, and test–retest equivalence for all raters were tested using the two-one-sided t-test procedure (TOST) [15]. This procedure relies on the calculation of the mean of the differences between measurements (either between test and retest results or between the results of the two advanced and the two beginner raters, respectively) and the associated 90% confidence intervals. The confidence intervals are then compared against pre-defined equivalence boundaries, which were set to ± 1 mm (i.e., one-third of the 3 mm cut-off value proposed as an indicator for ACL deficiency [17]) in our study. Measurements obtained by different examiners or at different test times were considered “equivalent”, if the 90% confidence intervals on both sides were found to lie fully within the above-mentioned boundaries of ± 1 mm. If confidence intervals were partly in- and partly outside the equivalence range, measurements were considered “inconclusive”, whereas confidence intervals lying fully outside the boundaries were termed as “nonequivalent”.

To warrant adequate statistical power of equivalence tests, the number of subjects to be included was determined through a priori power analysis. The calculation was based on an assumed standard deviation of differences in SSD of 1 mm, the above-mentioned equivalence boundaries of ± 1 mm, a two-sided type I error rate of *α* = 0.025 and the desired power 1-β = 0.8. This yielded the required sample size of 11 participants to be subject to repeated measurements. For the execution of equivalence tests and power analysis, the R TOSTER (v. 0.3.4) and PowerTOST (v. 1.5–2) packages were used [15].

Between-test differences were also used to calculate standard errors of measurement (*SEM*) as $SEM={SD}_{Diff}\bullet {\sqrt{2}}^{-1}$, where *SD*_*Diff*_ is the standard deviation of difference scores [12].

Intraclass correlation coefficients were calculated using two-way mixed effects models for absolute agreement of measurements between test days or raters. To facilitate the comparison of the test–retest reliability of all arthrometers, ICCs and SEMs were additionally calculated using pooled data acquired by all four raters. Negative ICCs, which may result in small samples as a consequence of the between-subjects variance being greater than the within-subjects variance, were considered “not reliable” (NR) and reported as such[16]. In addition to ICCs, Bland Altman plots were created to visualize the agreement of ratings both within and between raters.

In accordance with the recommendations by Koo and Li, ICCs were interpreted as poor when below 0.50, as moderate when between 0.50 and 0.75, as good when between 0.75 and 0.90 and as excellent when above 0.90[14].

## Results

A total of 2,304 Lachman Tests were performed. No test had to be stopped because of pain or discomfort.

### Inter-rater reliability

All reliability statistics reflecting the agreement of ATT and SSD measures obtained by pairs of advanced and beginner raters with all four arthrometer as well as corresponding measurement values are shown in Table 1.

**Table 1:** Inter-rater equivalence and reliability for Anterior Tibial Translation (ATT) and Side-to-Side Differences (SSD) measurements between advanced raters and beginners

	Anterior Tibial Translation (ATT)					Side-to-Side Difference (SSD)				
	Examiner 1	Examiner 2	*p*^¢^	ICC (95% CI)	SEM	Examiner 1	Examiner 2	*p*^¢^	ICC (95% CI)	SEM
Rolimeter	6.2 ± 1.5	6.5 ± 1.2	0.01	0.76* (0.37–0.92)	0.68	− 0.5 ± 1.1	− 0.1 ± 0.8	0.03	NR	1.15
KLT	6.9 ± 1.9	6.5 ± 1.5	n.s	0.72* (0.31–0.91)	0.90	− 0.5 ± 1.6	− 0.2 ± 0.8	0.09	0.25 (0–0.71)	1.14
KiRA	11.1 ± 1.4	11.3 ± 2.2	n.s	0.18 (0–0.68)	1.70	− 2.1 ± 1.6	− 0.2 ± 2.8	n.s	0.08 (0–0.55)	2.15
KT1000	8.5 ± 1.8	8.0 ± 2.2	n.s	0.70* (0.27–0.90)	1.08	− 1.3 ± 1.0	0.6 ± 1.3	n.s	0.16 (0–0.56)	0.89

	Examiner 3	Examiner 4	*p*^¢^	ICC (95% CI)	SEM	Examiner 3	Examiner 4	*p*^¢^	ICC (95% CI)	SEM
Rolimeter	4.8 ± 1.1	5.6 ± 1.1	n.s	0.51* (0–0.83)	0.89	0.3 ± 1.0	0.3 ± 1.1	< 0.01	0.83* (0.50–0.94)	0.46
KLT	5.5 ± 1.5	4.9 ± 1.3	n.s	0.49* (0–0.81)	1.00	0.4 ± 1.6	0.2 ± 1.5	< 0.01	0.54* (0–0.85)	1.07
KiRA	12.2 ± 3.8	14.5 ± 2.3	n.s	NR	3.90	−0.5 ± 2.5	2.9 ± 1.7	n.s	0.13 (0–0.51)	1.83
KT1000	7.6 ± 1.9	6.8 ± 1.6	n.s	0.64* (0.16–0.88)	0.95	0.2 ± 1.4	0.1 ± 1.1	n.s	0.38 (0–0.77)	1.01

All values obtained from retest. Mean ATTs ± standard deviation displayed for the left knee. SSD’s obtained by subtracting the value of the left from the right leg. ¢, significance level reported for the equivalence test with ± 1 mm equivalence boundary

*Significant ICCs (*p* < 0.05) are flagged with *. Standard errors of measurement (SEM) are given in mm

*NR* Not reliable; *ICC (95% CI)* Intraclass correlation (95% Confidence Interval

Examiner 1–2 = advanced rater; Examiner 3–4 = beginner

For ATT testing, ICCs showed “good” agreements only for advanced raters using Rolimeter. With all other devices, the agreement between testers was “poor” to “moderate” (Table 1). For SSD data, ICCs reflected generally “poor” conformity (except for “good” and “moderate” agreement with Rolimeter and KLT between examiner 3 and 4, respectively) between raters (Table 1). SEM for ATT and SSD ratings were lowest for the Rolimeter and highest for KiRA (Table 1).

#### Equivalence testing

**Fig. 2:**
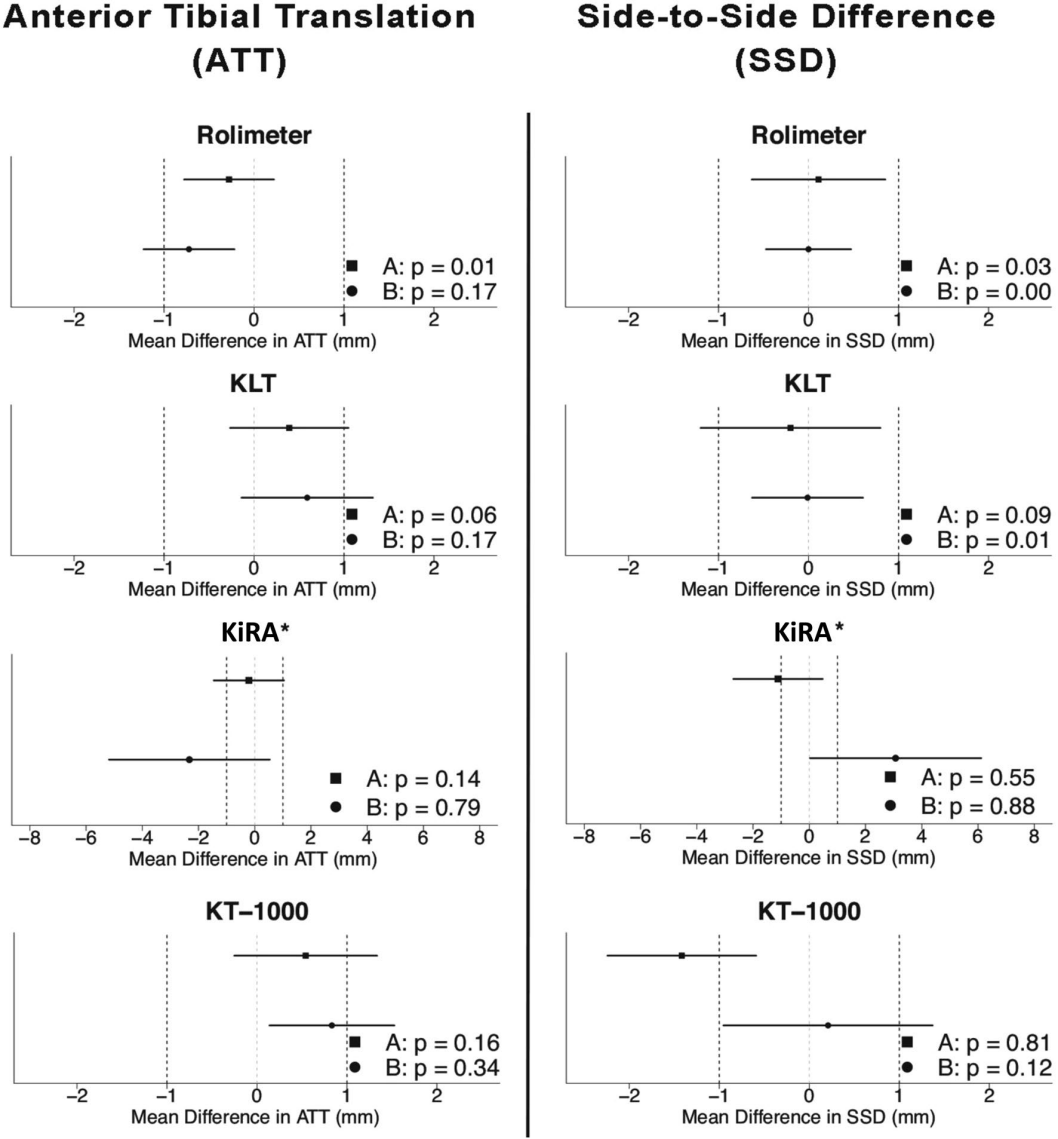
Inter-rater equivalence testing for Anterior Tibial Translation (ATT) and Side-to-Side Difference (SSD) in healthy individuals for all arthrometers between advanced (**a**) and beginner (**b**) raters. Equivalence boundariesy’s are set to ± 1 mm. * Please note the different scaling (± 6 mm) for KiRA arthrometer

The only arthrometer to yield equivalent ATT measurements of different raters was the Rolimeter, when used by advanced raters (Table 1, Fig. 2). For SSD measurements, equivalent results were obtained with the Rolimeter by both advanced raters (examiner 1 and 2) and beginners (examiner 3 and 4) and KLT by beginners only. All other equivalence test results failed to reach statistical significance and were considered as “inconclusive” (Table 1, Fig. 2).

### Intra-rater reliability

Intra-rater reliability results showing the agreement of repeated measures by examiner and device are shown in Table 2. Depending on device and rater experience, ICCs indicated “good” to “excellent” agreement of ATT, and “moderate” to “good” agreement of SSD measurements. Intra-rater reliability was generally slightly higher for advanced raters compared to beginners (Table 2). Pooled data from all four raters summarizing the respective reliability statistics calculated to quantify the agreement of test and retest measures of ATT and SSD are presented in Table 3. Just as for tests of inter-rater reliability, SEMs for ATT and SSD ratings were lowest for the Rolimeter and highest for KiRA.

**Table 2:** Intra-rater equivalence and reliability for Anterior Tibial Translation (ATT) and Side-to-Side Differences (SSD) measurements for each rater

	Anterior Tibial Translation (ATT)															
	Rolimeter				KLT				KiRA				KT-1000			
	Mean ± SD	*p*^¢^	ICC (95% CI)	SEM	Mean ± SD	*p*^¢^	ICC (95% CI)	SEM	Mean ± SD	*p*^¢^	ICC (95% CI)	SEM	Mean ± SD	*p*^¢^	ICC (95% CI)	SEM
Ex 1Test	5.8 ± 1.4	< 0.01	0.94 (0.25–0.99)	0.23	6.9 ± 1.9	< 0.01	0.87 (0.60–0.96)	0.73	11.1 ± 1.9	0.02	0.63 (0.10–0.88)	1.04	8.5 ± 2.0	< 0.01	0.91 (0.73–0.97)	0.57
Ex 1 Retest	6.2 ± 1.5				6.9 ± 1.9				11.1 ± 1.4				8.5 ± 1.8			
Ex 2 Test	6.3 ± 1.1	< 0.01	0.92 (0.76–0.98)	0.31	6.6 ± 1.3	< 0.01	0.90 (0.70–0.97)	0.45	11.5 ± 2.0	0.03	0.79 (0.41–0.93)	1.00	8.2 ± 2.1	< 0.01	0.94 (0.80–0.98)	0.53
Ex 2 Retest	6.5 ± 1.2				6.5 ± 1.5				11.3 ± 2.2				8.0 ± 2.2			
Ex 3 Test	5.1 ± 1.4	< 0.01	0.89 (0.67–0.97)	0.38	5.6 ± 1.6	< 0.01	0.89 (0.66–0.97)	0.73	12.3 ± 4.0	n.s	0.68 (0.18–0.90)	2.27	7.9 ± 1.6	0.03	0.77 (0.40–0.93)	0.83
Ex 3 Retest	4.8 ± 1.1				5.5 ± 1.5				12.2 ± 3.8				7.6 ± 1.9			
Ex 4 Test	5.5 ± 0.9	< 0.01	0.72 (0.27–0.91)	0.55	5.0 ± 1.2	< 0.01	0.83 (0.50–0.95)	0.54	15.0 ± 2.8	n.s	0.36^$^ (0–0.76)	2.05	6.7 ± 1.7	< 0.01	0.89 (0.66–0.97)	0.56
Ex 4 Retest	5.6 ± 1.1				4.9 ± 1.3				14.5 ± 2.3				6.8 ± 1.6			

	Side-to-side difference (SSD)															
	Rolimeter				KLT				KiRA				KT-1000			
	Mean ± SD	*p*^¢^	ICC (95% CI)	SEM	Mean ± SD	*p*^¢^	ICC (95% CI)	SEM	Mean ± SD	*p*^¢^	ICC (95% CI)	SEM	Mean ± SD	p^¢^	ICC (95% CI)	SEM
Ex 1 Test	0.7 ± 0.7	< 0.01	0.77 (0.25–0.93)	0.42	1.7 ± 1.3	0.02	0.76 (0.34–0.92)	0.97	2.3 ± 1.8	n.s	0.21^$^ (0–0.69)	1.84	1.5 ± 0.8	< 0.01	0.35^$^ (0–0.76)	0.85
Ex 1 Retest	0.9 ± 0.7				1.3 ± 1.0				2.3 ± 1.2				1.4 ± 0.7			
Ex 2 Test	0.7 ± 0.7	< 0.01	0.91 (0.70–0.97)	0.29	0.6 ± 0.4	< 0.01	0.66 (0.16–0.89)	0.46	1.9 ± 1.3	0.06	0.84 (0.53–0.95)	1.04	1.0 ± 1.1	0.07	0.66 (0.20–0.89)	0.77
Ex 2 Retest	0.7 ± 0.5				0.7 ± 0.5				2.1 ± 1.8				1.0 ± 1.0			
Ex 3 Test	0.8 ± 0.6	< 0.01	0.71 (0.24–0.91)	0.54	1.1 ± 0.8	0.02	0.77 (0.40–0.93)	0.97	3.3 ± 1.7	n.s	0.51 (0–0.83)	2.29	1.3 ± 0.7	0.02	0.49 (0–0.82)	1.05
Ex 3 Retest	0.8 ± 0.6				1.3 ± 1.0				2.2 ± 1.1				1.0 ± 1.0			
Ex 4 Test	0.6 ± 0.7	< 0.01	0.42^$^ (0–0.79)	0.76	0.8 ± 0.6	< 0.01	0.66 (0.14–0.89)	0.77	4.1 ± 3.6	n.s	NR	3.85	1.0 ± 0.8	< 0.01	0.77 (0.40–0.93)	0.57
Ex 4 Retest	0.9 ± 0.7				1.1 ± 1.0				2.9 ± 1.7				0.9 ± 0.6			

All SSD measurements obtained during retest. ATT measurements obtained from the left knee. Measurements displayed as mean ± standard deviation (SD),

^¢^Significance level reported for the equivalence test with ± 1 mm equivalence boundary. All ICCs are significant (*p* < 0.05) if not flagged with § (*p* > 0.05)

Standard errors of measurement (SEM) are given in millimeter (mm)

*NR* not reliable; *Ex* Examiner; *ICC (95% CI)* Intraclass correlation (95% Confidence Interval)

Examiner 1–2 = advanced rater; Examiner 3–4 = beginners

**Table 3:** Measures of intra-rater reliability by arthrometer based on pooled data from all four examiners

	Anterior tibial translation (ATT)			Side–to–side difference (SSD)		
	Mean ± SD	ICC	SEM	Mean ± SD	ICC	SEM
Rolimeter	5.8 ± 1.4*	0.9	0.41	0.8 ± 0.6*	0.7	0.54
KLT	6.0 ± 1.7*	0.9	0.56	1.1 ± 0.9*	0.73	0.74
KiRA	12.3 ± 2.8	0.7	1.64	2.4 ± 1.5	0.11	3.03
KT-1000	7.7 ± 1.9	0.89	0.63	1.1 ± 0.9*	0.66	0.81

All SSD measurements obtained during retest. ATT measurements obtained from the left knee during retest

Standard errors of measurement (SEM) are given in mm

*Ex* Examiner

*Equivalent measurements between arthrometers

#### Equivalence testing

**Fig. 3:**
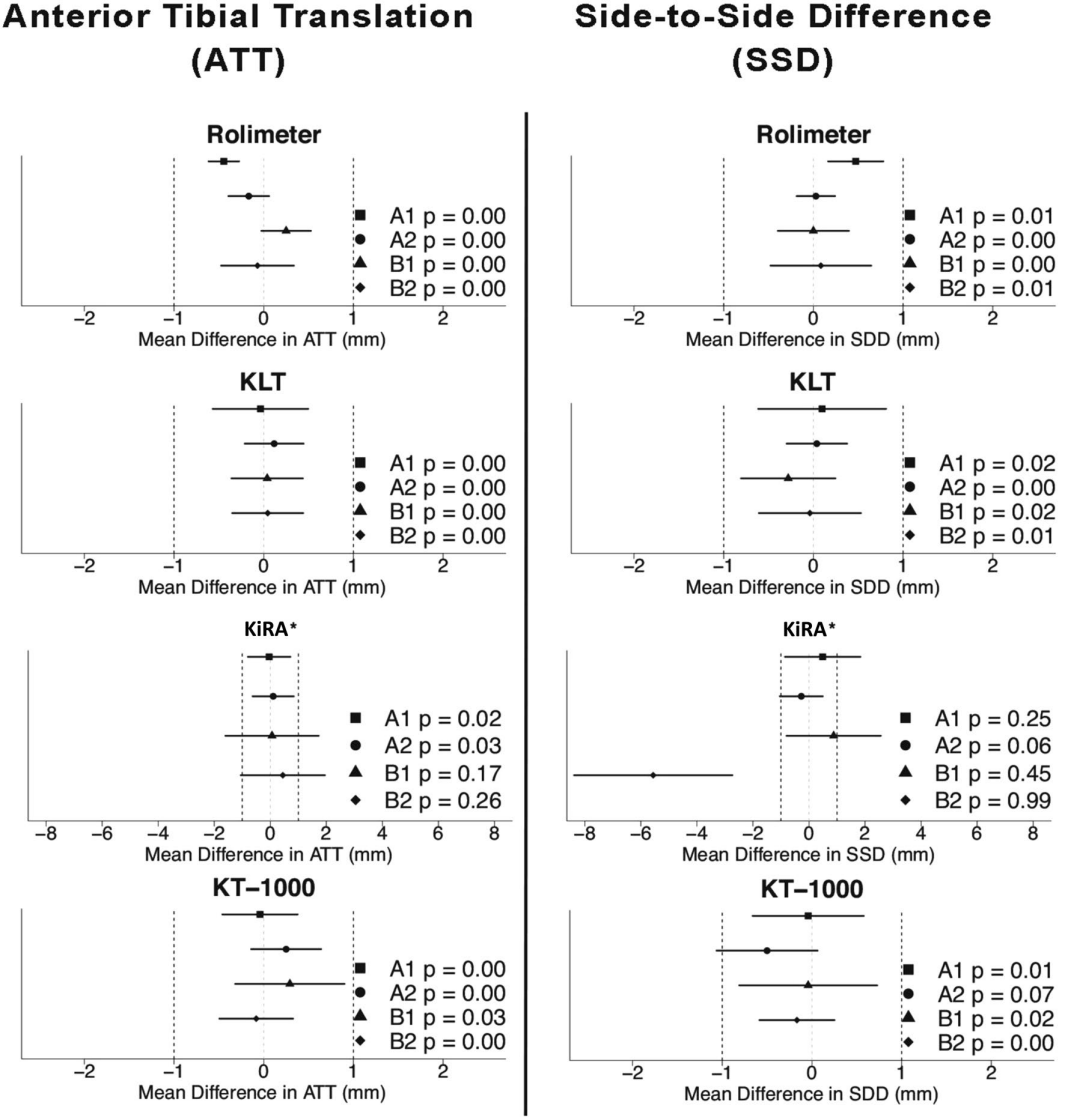
Intra-rater equivalence testing for Anterior Tibial Translation (ATT) and Side-to-Side Difference (SSD) in healthy individuals for all arthrometers between all four raters (A1, A2 = advanced raters; B1, B2 = beginner) Equivalence boundary’s are set to ± 1 mm. * Please note the different scaling (± 8 mm) for KiRA arthrometer

Using the Rolimeter, KLT and KT-1000, all raters achieved equivalent test–retest results of ATT and SSD measurements, except for the KT1000 measurement of examiner 2, which showed an “inconclusive” result (Table 2, Fig. 3). With KiRA, TOST test results were “inconclusive” for ATT measurements when performed by beginners and for SSD measurements in examiners 1–3 (Table 2, Fig. 3). The agreement of ratings with KiRA was higher in examiner 1 and 2 (advanced testers) compared to examiner 3 and 4 (beginners) (Table 2).

Bland Altman plots, visualizing the agreement of ratings both within and between raters are provided as supplementary material.

### Ratios of false positive results

Inspection of the 96 SSDs calculated for each device showed no false positive results (SSD > 3 mm) with the Rolimeter (0%), two (2.1%) with the KT-1000 and KLT and 33 (34.4%) with KiRA arthrometer, respectively. False positives were more common in beginners (23, 24.0%) than in experienced raters (10, 10.4%).

## Discussion

The main finding of the present study was that intra-rater reliability of arthrometer measurements is acceptable with Rolimeter, KLT and KT-1000, whereas inter-rater reliability is generally poor with all tested devices. Standard errors of measurements (SEM), absolute anterior tibial translation (ATT) as well as side-to-side differences (SSD) in ATT are comparable between Rolimeter, KLT and KT-1000 but higher for KiRA.

To allow for results obtained by different examiners, at different times and with different devices to be directly compared, adequate inter- and intra-rater reliabilities as well as the knowledge about the equivalence of measurements between arthrometers are required. In the present study, the inter- and intra-rater reliability of the above arthrometers was tested using the TOST procedure (to assess the equivalence of test results), ICCs (quantifying the agreement of results) and SEMs (reflecting typical measurement errors). While already established method endorsed by the US Food and Drug Administration (FDA) and European Medicines Agency (EMA) [22], equivalence testing has only just started expanding into the fields of surgery and orthopedics. The TOST procedure tests the proper null hypothesis that measurement results are nonequivalent and may, in case of significant test results, provide true evidence of equivalence, rather than just the lack of evidence for statistical difference that is usually reported (e.g., non-significant t-tests or ANOVAs) [7, 22]. While equivalence tests are the only useful comparative statistics to be applied in reliability studies (lack of difference may easily be provoked by including fewer participants), it is important to understand that they rely on the definition of a range in which measurement results are similar enough to be considered clinically equivalent [23]. In our study, we decided to set these equivalence boundaries to ± 1 mm based on the following considerations: First, 1 mm represents one-thirdone third of the 3 mm cut-off value that has been proposed as an indicator of ACL deficiency [17]; and second, the range appears reasonably dimensioned when compared to the typical errors examiners made with the same arthrometers in repeated measures (0.41 mm < SEM < 0.81 mm).

The results of the present study suggest that in repeated measures (intra-rater), equivalent results of both ATT and SSD can be obtained with the Rolimeter, KLT and KT-1000 arthrometers but not consistently with KiRA. ICCs varied between arthrometers and examiners and ranged between 0.36–0.94 for ATT and 0.21–0.91 for SSD measurements. With the Rolimeter, KLT and KT-1000 SEMs were typically smaller than 1 mm for both ATT and SSD and of similar dimension, irrespective of the arthrometer used. With KiRA, by contrast, SEMs were substantially larger (1.04–3.85 mm), which lends support to our observation that test–retest results with this arthrometer are nonequivalent.

While the joint statistical analyses of ATT and SSD values suggest that intra-rater reliability is acceptable with three (Rolimeter, KLT, KT1000) out of the four arthrometers tested, measures of inter-rater reliability clearly showed that results obtained by different examiners are not readily comparable. For SSD, equivalent test results were found with the Rolimeter (between both advanced and beginner raters) and KLT (between beginners only). Considering also the ICCs, which mostly showed poor agreement, particularly of SSD ratings, our data warrant caution in comparing results obtained by different examiners. Several factors may explain the low ICCs and conflicting results between raters. In addition to statistical reasons (ICCs relate the between-subject to the within-subject variance, with the former being typically small, particularly for SSD measures in healthy subjects), inconsistent positioning of patient and device might lead to strongly deviating measurements. Moreover, measures of ATT may also be affected by differences in the forces applied during the execution of the Lachman test, which is why only the usage of SSD values is recommended in clinical routine.

The results of the present study conflict with earlier reliability studies to report both high intra- and inter-rater reliability, but are in line with more recent investigations. Klasan et al. reported both a significant device- and investigator effect in KT-1000 laxity testing of 770 healthy knees by 24 different investigators with similar experience [13]. The intra-class ICCs ranged from “not reliable” to “excellent” and jointly showed a moderate agreement of results [13]. Similarly, Wiertsema et al. reported ICC values of 0.47 and 0.14 for intra- and inter-rater reliability between two testers, respectively [24].

While adequate inter- and intra-rater reliability is important in the field of science and research, in daily practice, it is particularly a low rate of false positivefalse-positive measurements that is of primary importance. The rates of false positives have previously been documented for the KT-1000 and Rolimeter and ranged between 2- and 5% [2, 9, 17]. However, no respective data have been published for KLT and KiRA. In the present investigation, the rate of false positive measurements was low for the Rolimeter (0%), KT-1000 (2.1%) and KLT (3.1%) but substantially higher for KiRA (34.4%). In beginners, measurements with an SSD greater than 3 mm were more frequently recorded (24.0%) as in more experienced raters (10.4%). The reasons for the differences in the reliability of measures obtained with KiRA and the other devices are speculative and discussed in the limitations section.

To summarize, our data testify to “good” to “excellent” and “moderate” to “good” test–retest reliability when measures of ATT and SSD are performed with the Rolimeter, KLT or KT-1000 arthrometers. Inter-rater reliability, by contrast, was inadequate with all arthrometers tested. It is, therefore, recommended that patients always be examined by the same investigators in repeated measurements. Furthermore, Rolimeter, KLT and KT-1000 yield results of comparable dimension for ATT and SSD values, whereas measures obtained with KiRA are substantially larger. In repeated tests, measurement reproducibility was only slightly higher in more experienced raters. Special care and increased experience are needed when using KiRA, since this device seems particularly sensitive to improper handling.

This study has some limitations. First, only healthy individuals with no prior knee injuries were examined. While obtaining baseline data in healthy subjects, in whom both knee laxity and, particularly, SSD values may be expected to be small, is important, further studies including ACL-injured and ACL-reconstructed patients are necessary to provide a more comprehensive picture, especially of the newly introduced and poorly studied arthrometers KLT and KiRA. Second, it must be pointed out that KiRA may provide live visual feedback during test administration. In the present study, all testers were blinded to any visual feedback in order to reduce bias and allow for comparisons between arthrometers to be made. This may have negatively biased the reliability data achieved with this device. Conversely, the test set up and handling of the KLT, Rolimeter and KT-1000 are similar, which may positively influence the respective reliabilities. Third, all participants were tested and retested on the same day. Consequently, our data may not allow for direct conclusions about between-day test–retest reliability to be drawn. Last, while advanced users had at least five years of clinical experience in manual knee examination, they only used one of the arthrometers on a regular basis. However, all examiners were given the opportunity to familiarize with all tested arthrometers prior to the beginning of the study.

## Conclusion

Intra-rater reliability in knee arthrometer testing is adequate (ATT: good to excellent; SSD: “moderate to good”) with three (Rolimeter, KLT, KT-1000) out the four devices tested. The inter-rater reliability, by contrast, is generally poor with all arthrometers (Rolimeter, KLT, KiRA, KT-1000). Knee laxity measures are comparable between the Rolimeter, KLT and KT-1000 but higher for KiRA. Clinically, the present results recommend that repeated measurements should always be performed by the same investigators.

## Supplementary Information

Below is the link to the electronic supplementary material.

Supplementary file1 (DOCX 525 KB)
