# Correction to: The evaluation of Rolimeter, KLT, KiRA and KT-1000 arthrometer in healthy individuals shows acceptable intra-rater but poor inter-rater reliability in the measurement of anterior tibial knee translation

**DOI:** 10.1007/s00167-021-06726-1

**Published:** 2021-09-14

**Authors:** Armin Runer, Tommaso Roberti di Sarsina, Vasco Starke, Alessandra Iltchev, Gernot Felmet, Sepp Braun, Christian Fink, Robert Csapo

**Affiliations:** 1 Department of Orthopaedics and Traumatology, Medical University of Innsbruck, Innsbruck, Austria; 2 Clinica Ortopedica e Traumatologica, Istituto Ortopedico Rizzoli, Bologna, Italy; 3 Medical University of Innsbruck, Innsbruck, Austria; 4 Orthopädische Praxis and ARTICO Sportklinik, Schwenningen, Germany; 5 Gelenkpunkt - Sports and Joint Surgery, Innsbruck, Austria; 6 Medical Informatics and Technology, ISAG, Research Unit for Orthopaedic Sports Medicine and Injury Prevention, Private University for Health Sciences, Innsbruck, Austria

## Correction to: Knee Surgery, Sports Traumatology, Arthroscopy (2021) 29:2717–2726 10.1007/s00167-021-06540-9

In the above-mentioned article there is an error stating that the Rolimeter (Aircast Europe) is no longer commercially available. This information is not correct. There are still ways to purchase the Rolimeter, including on the recently launched website www.rolimeter.com.

