# Supplementary material for: The evaluation of Rolimeter, KLT, KiRA and KT-1000 arthrometer in healthy individuals shows acceptable intra-rater but poor inter-rater reliability in the measurement of anterior tibial knee translation
Source: Knee Surg Sports Traumatol Arthrosc. 2021 Mar 31;29(8):2717–26. doi: 10.1007/s00167-021-06540-9 (PMC8298217; doi:10.1007/s00167-021-06540-9)
Supplement: Supplementary file 1 — Supplementary file1 (DOCX 525 KB) [file 167_2021_6540_MOESM1_ESM.docx]

| **Arthrometer** | **Inter-Rater Advanced** | **Inter-Rater Beginner** |
| --- | --- | --- |
| Rolimeter | 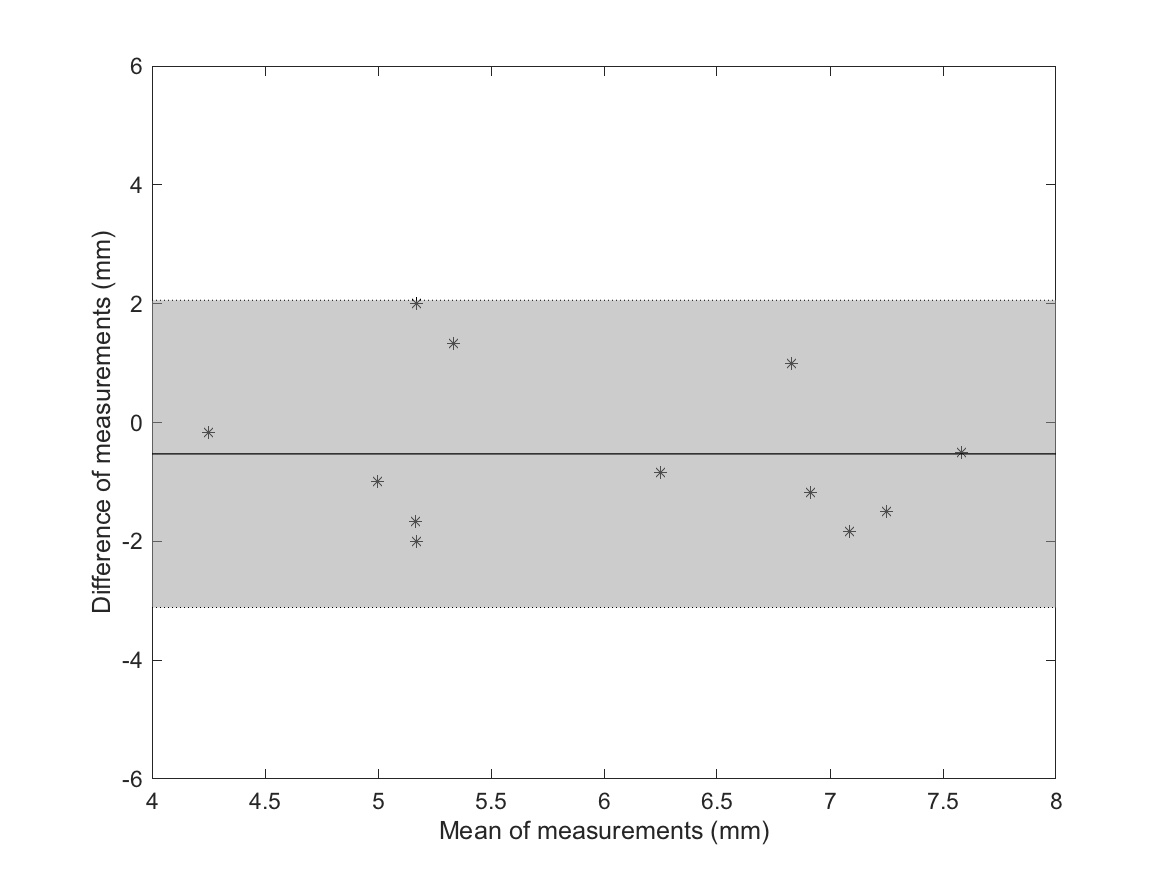 | 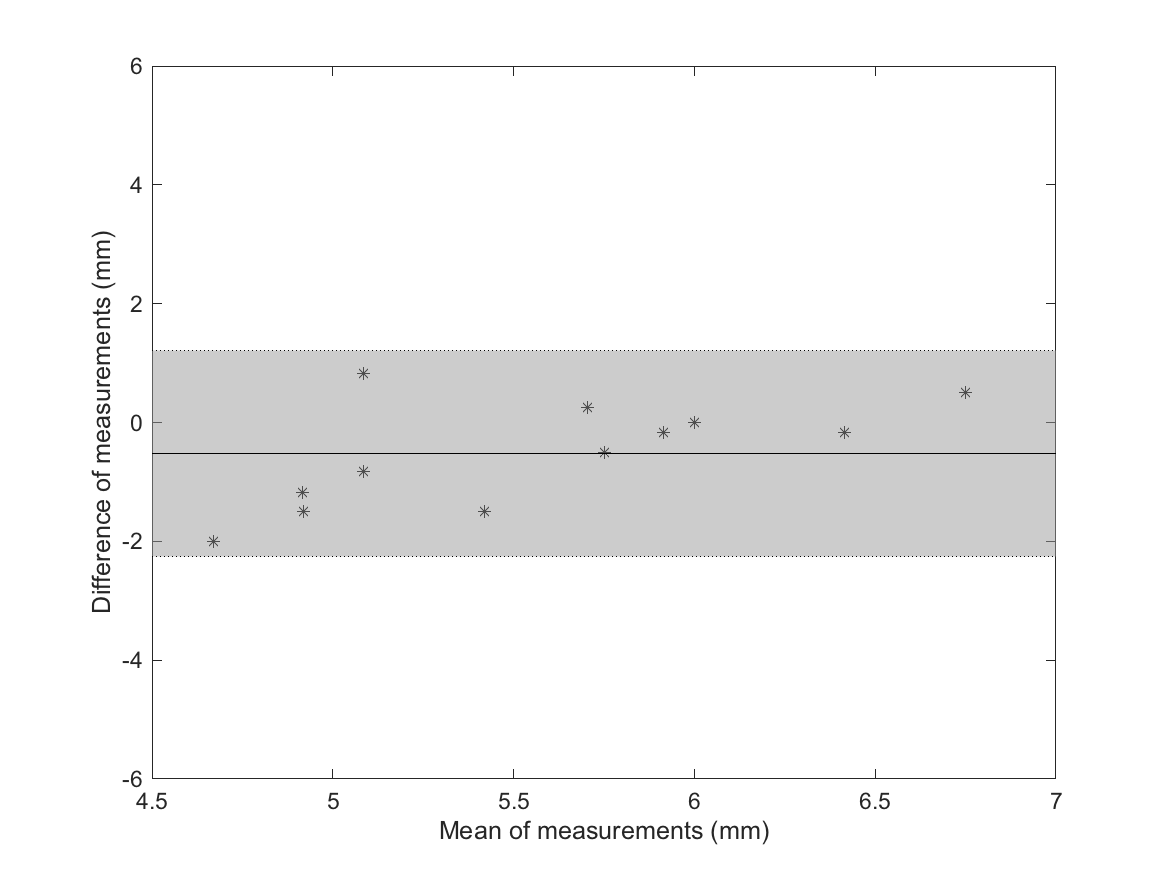 |
| KT1000 | 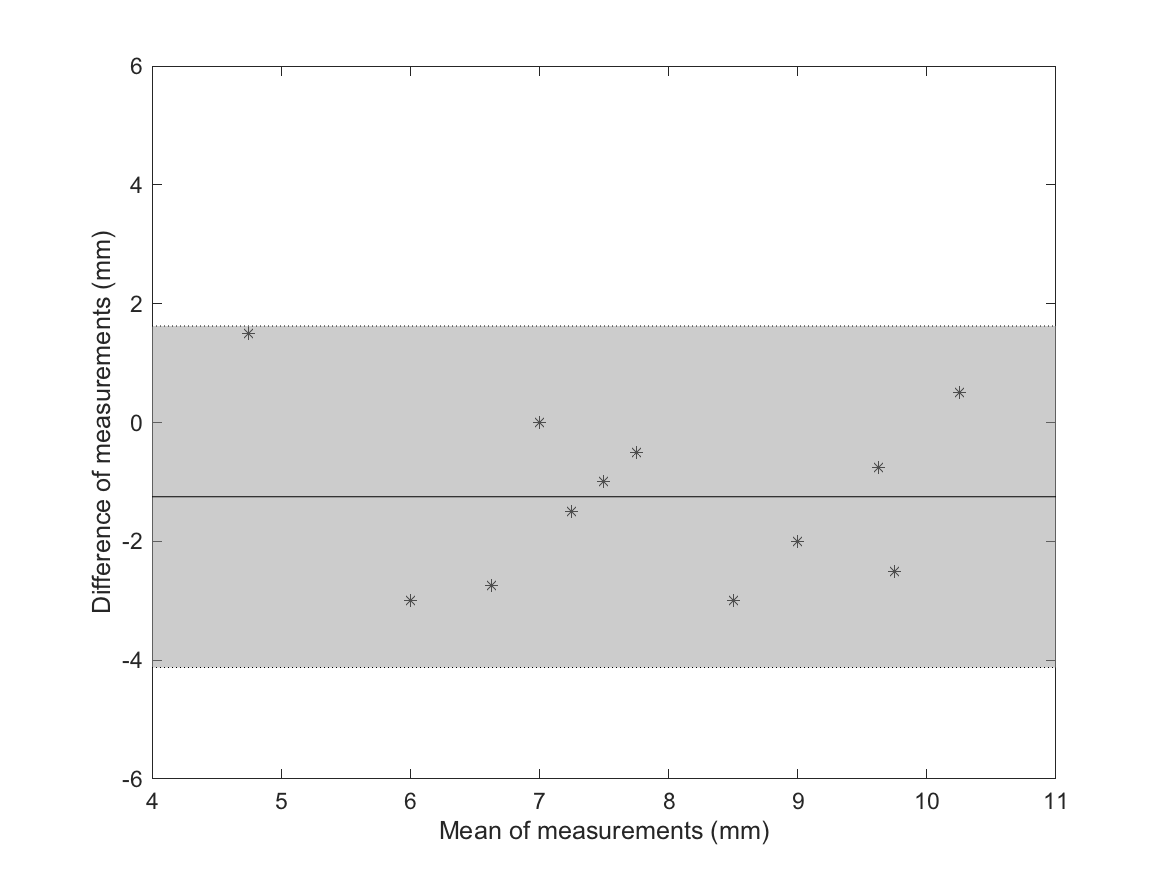 | 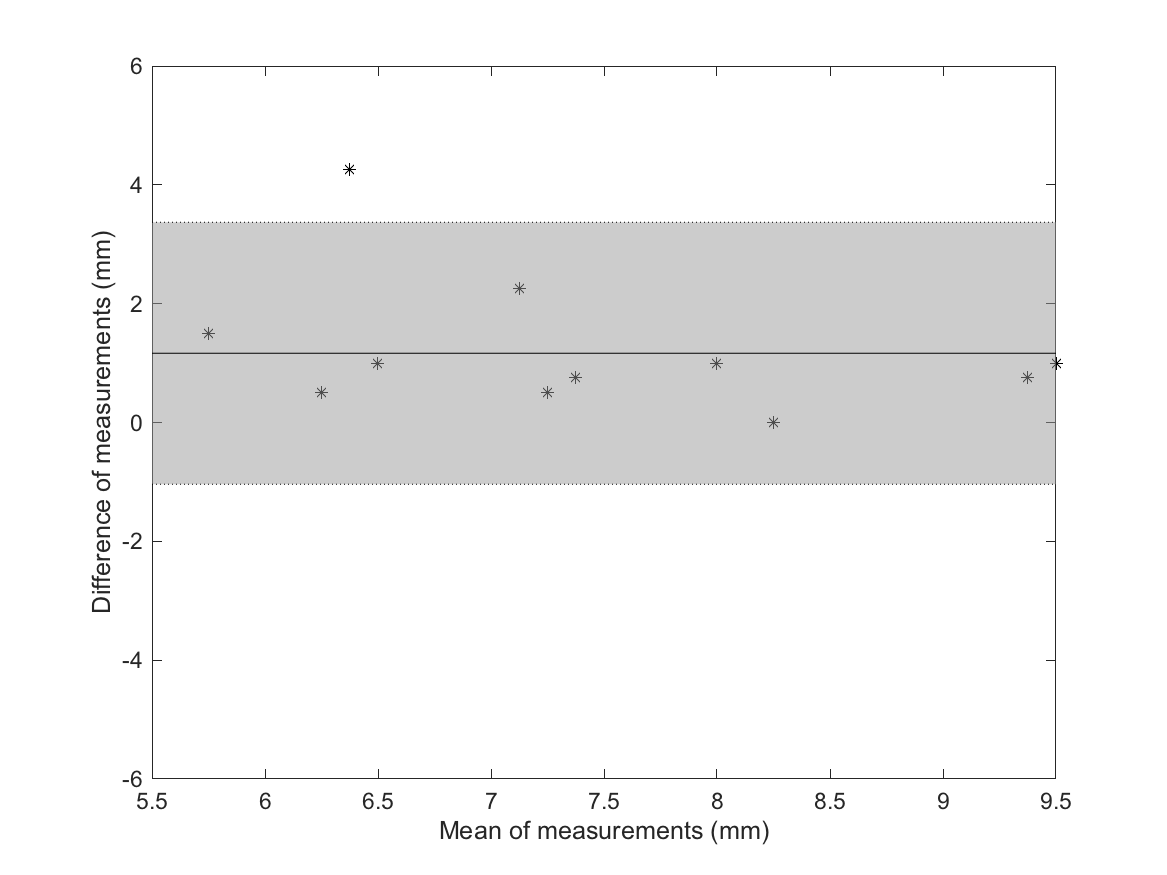 |
| KIRA | 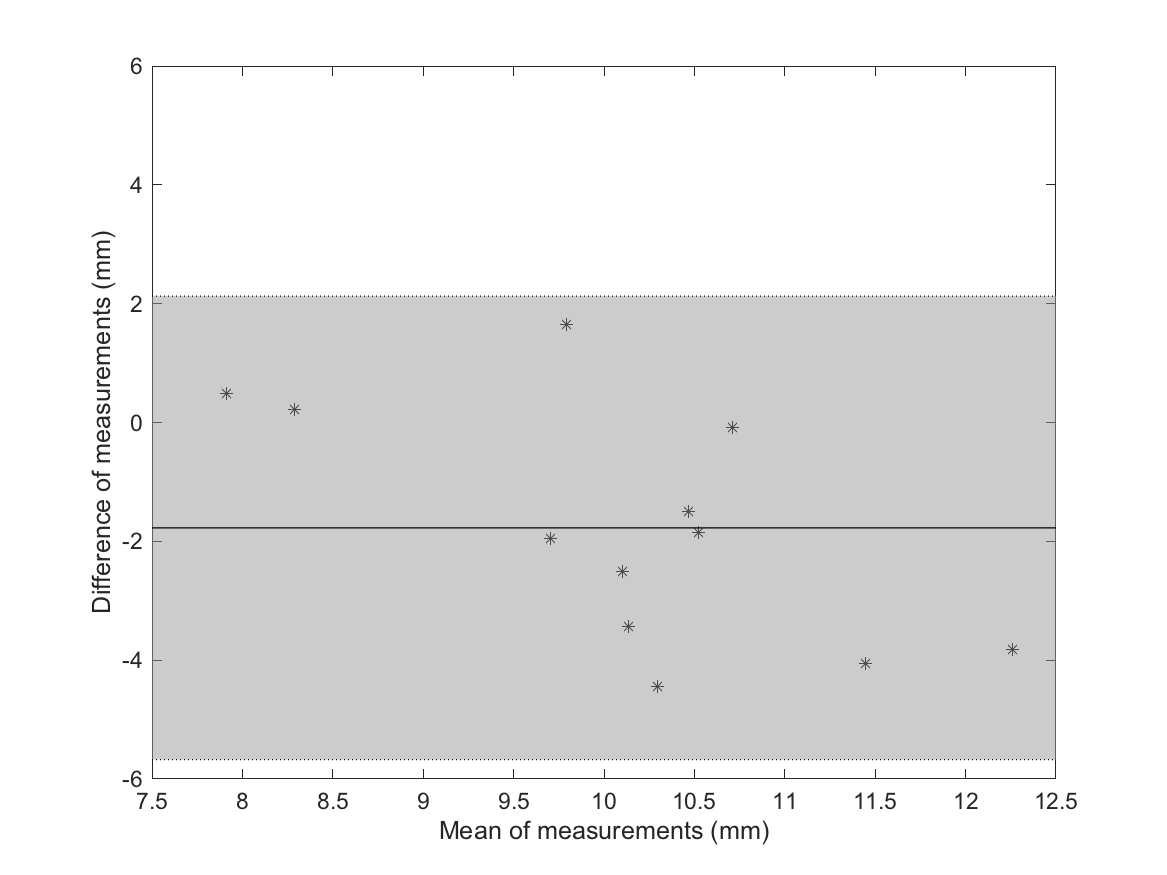 | 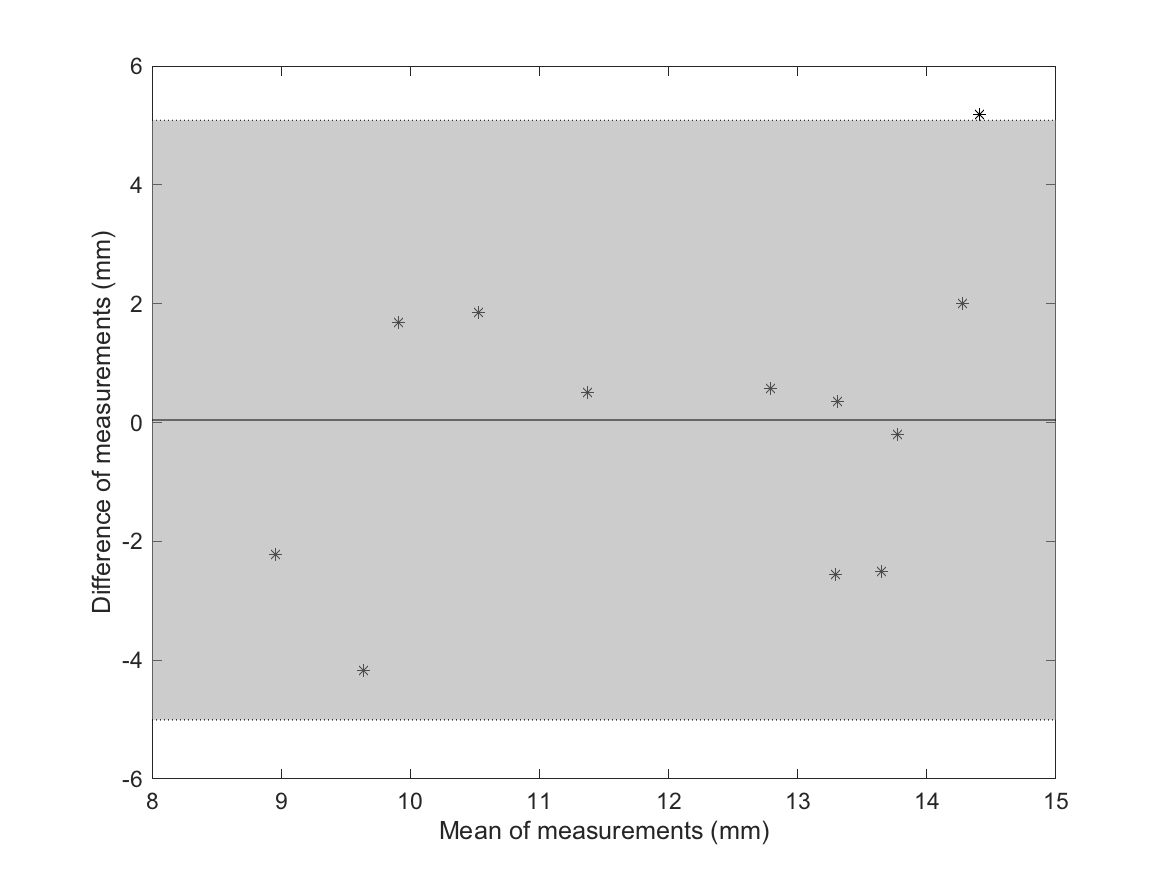 |
|  |  |  |
| KLT | 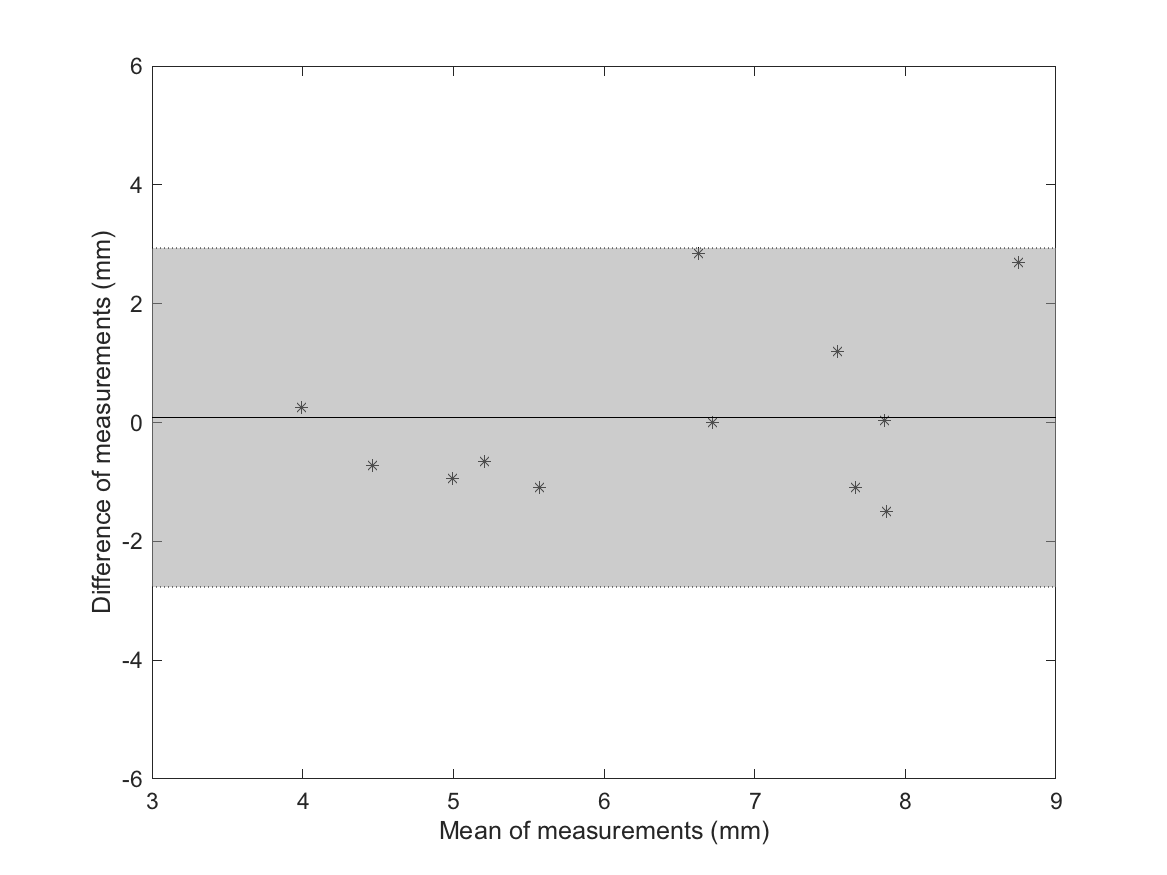 | 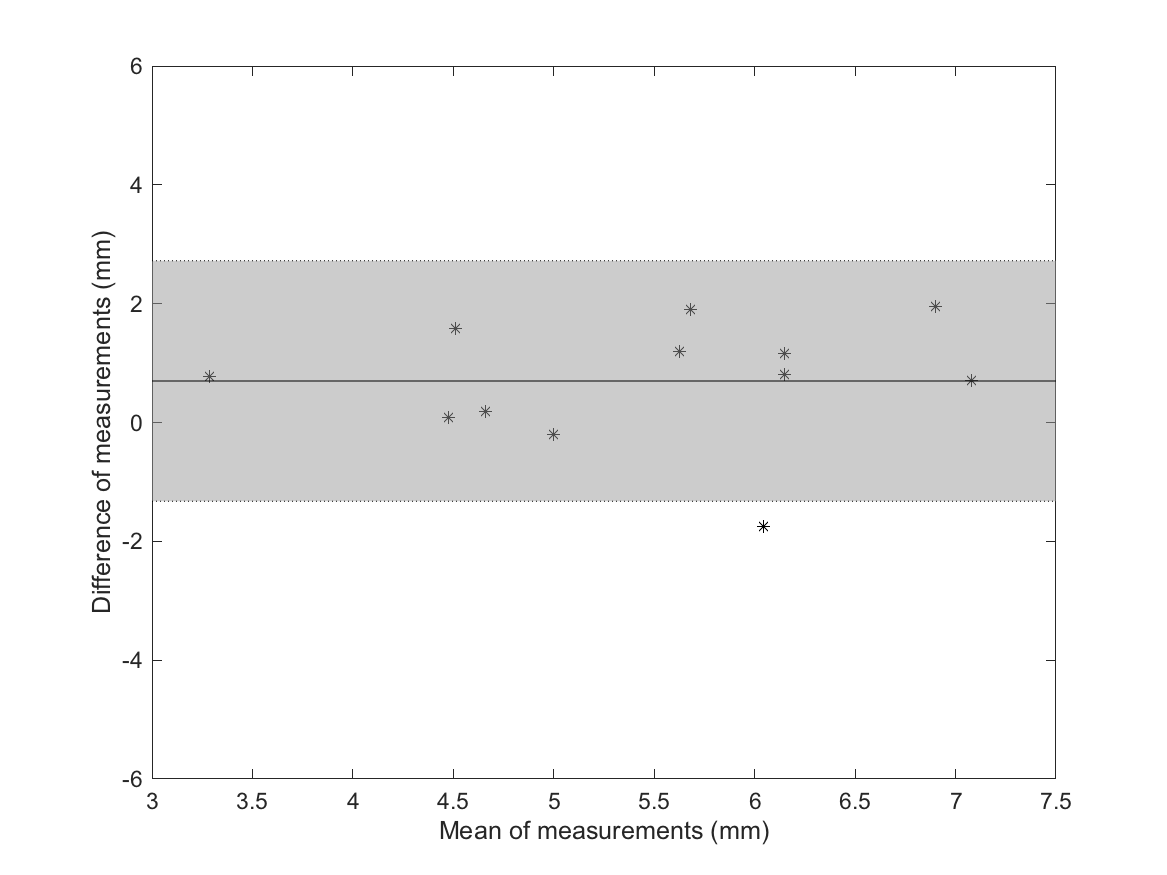 |

# Joint Bland-Altman plots for intra-rater comparisons

| **Arthrometer** | **Intra-rater Advanced** | **Intra-rater Beginner** |
| --- | --- | --- |
| Rolimeter | 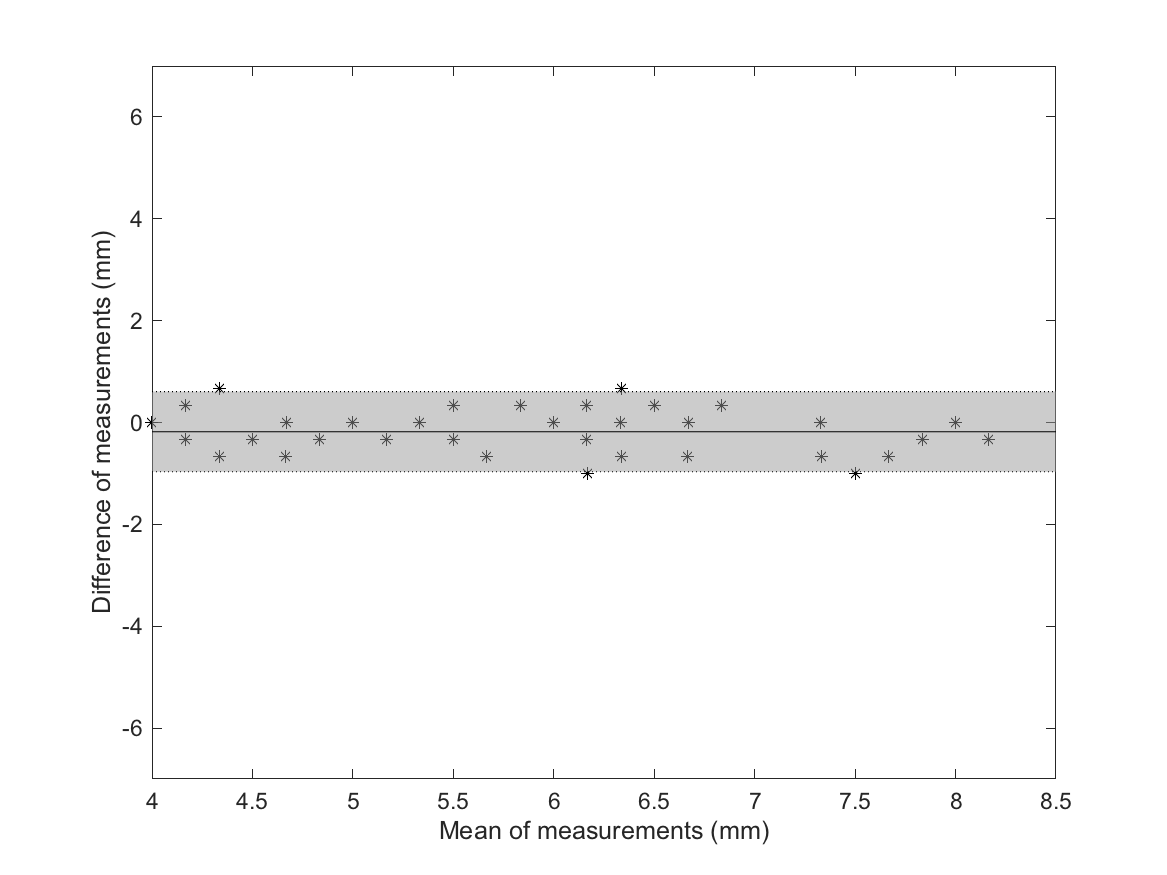 | 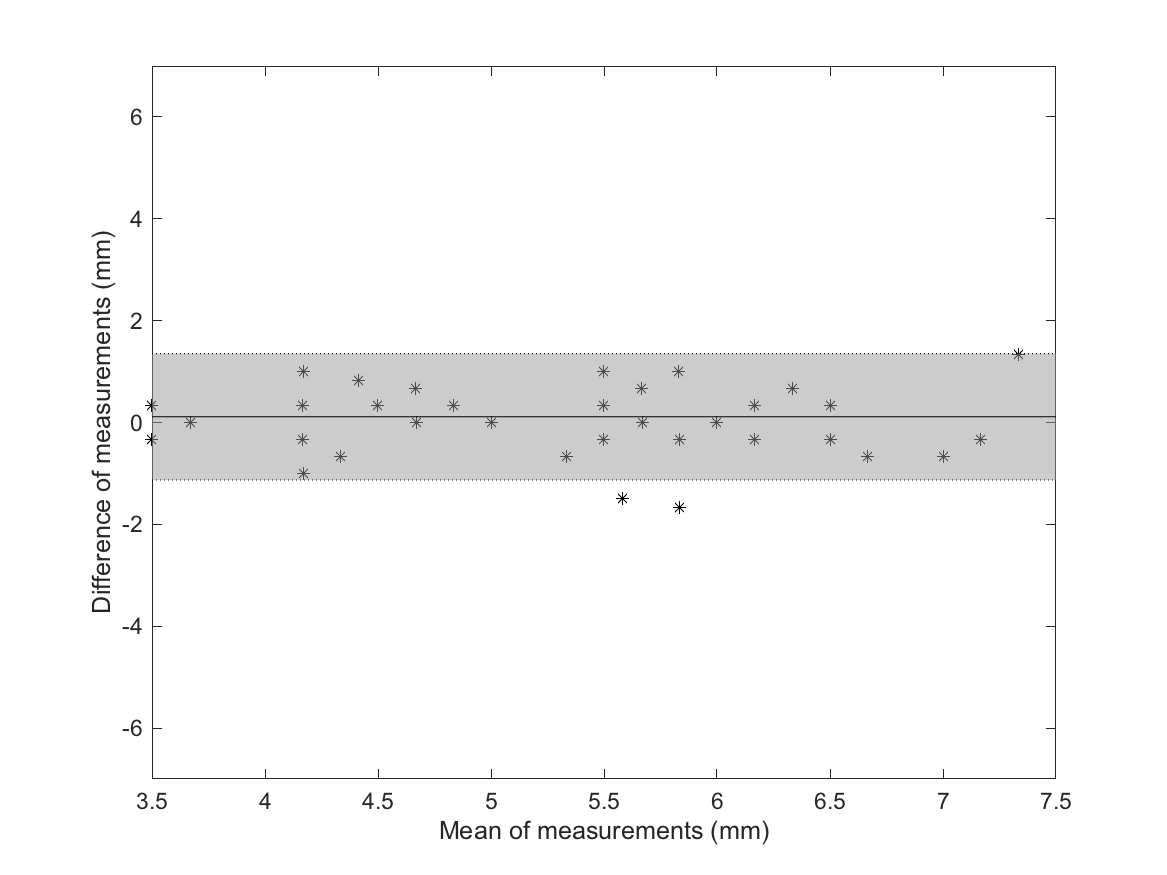 |
| KT1000 | 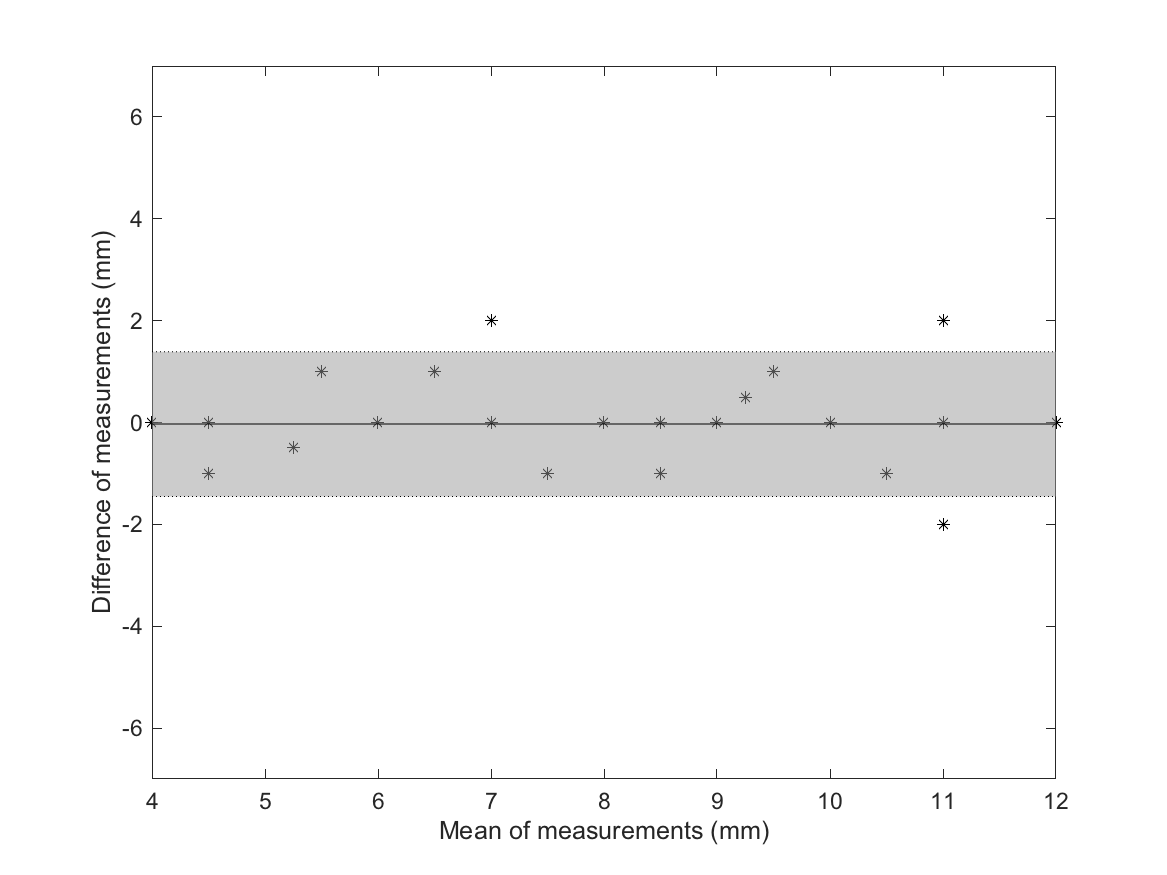 | 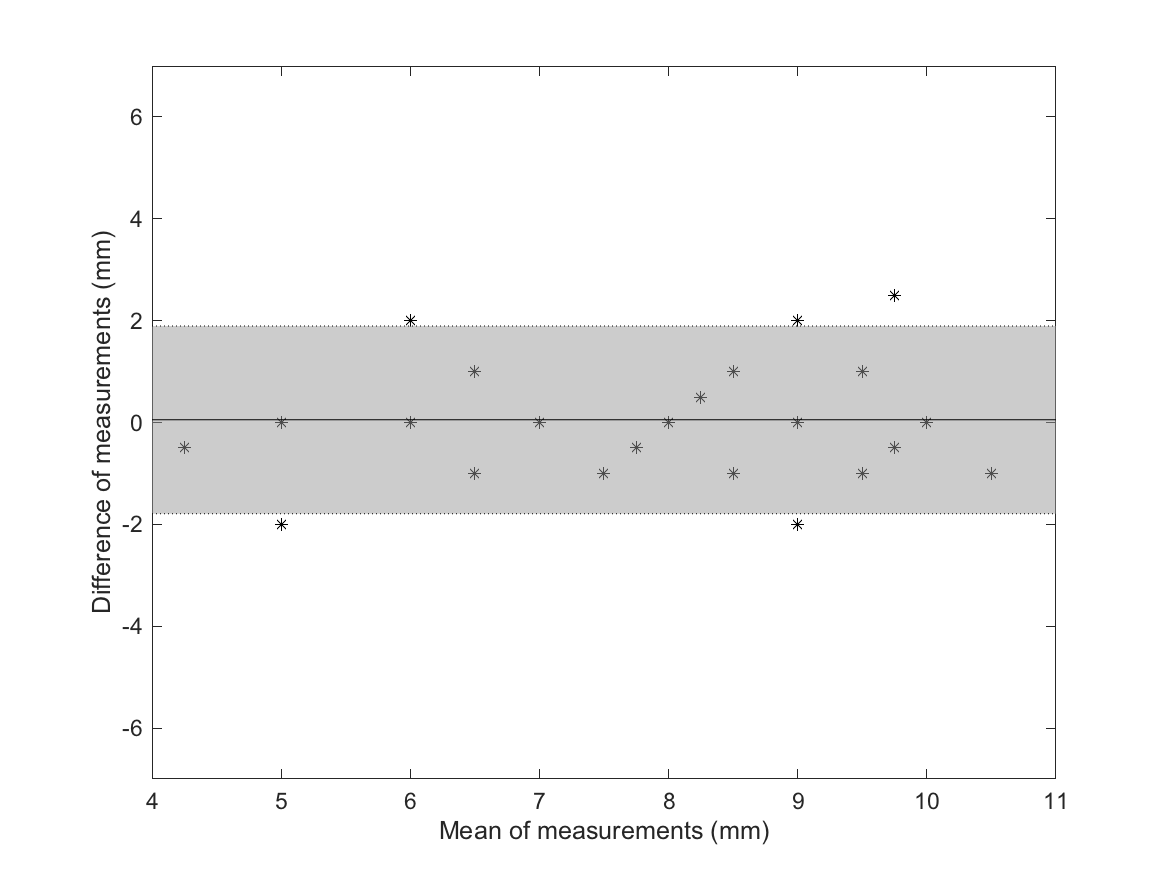 |
| KIRA | 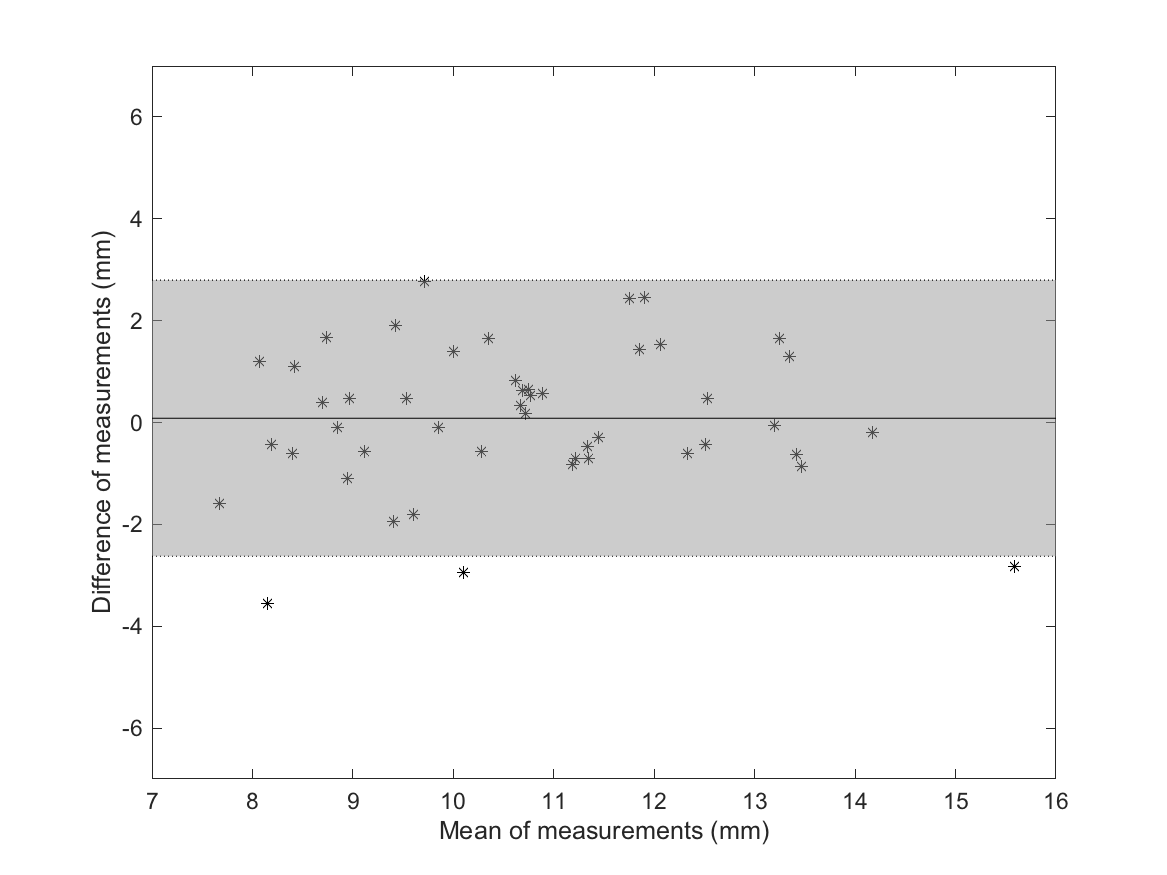 | 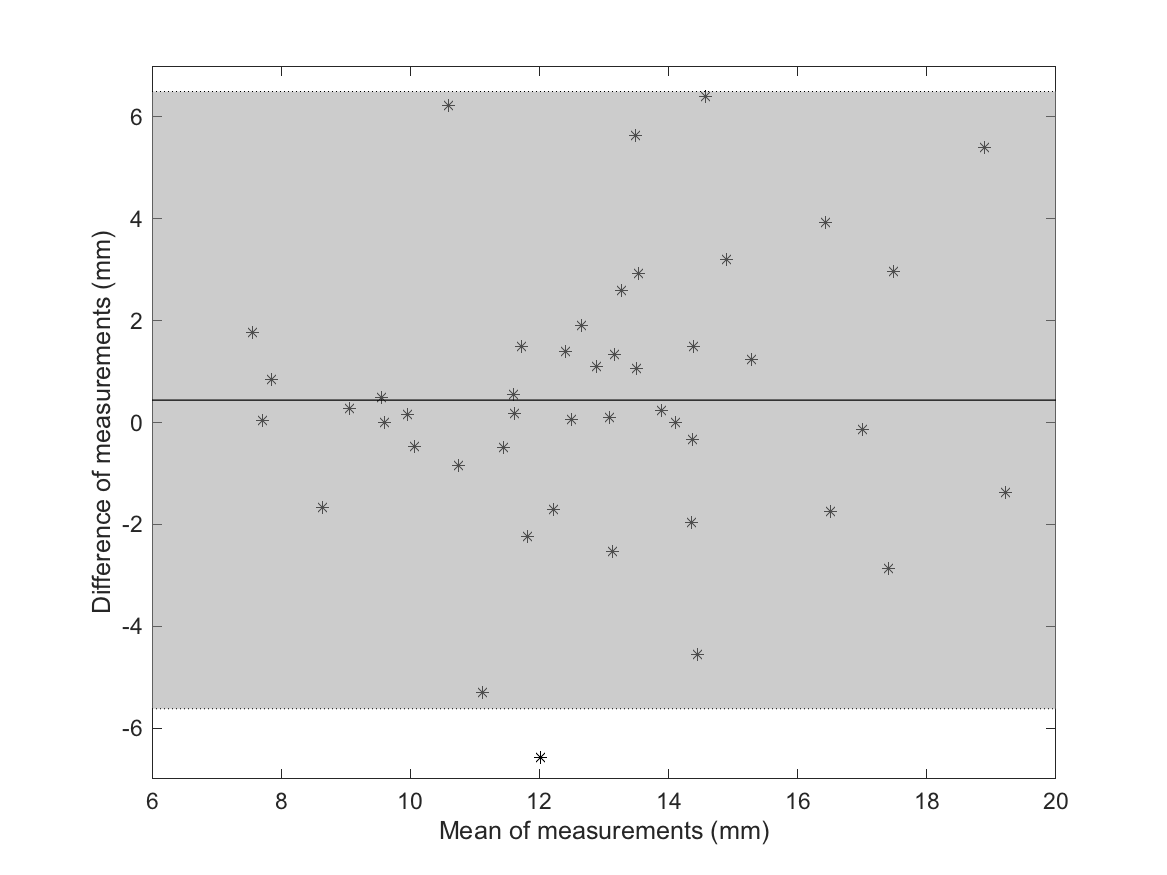 |
|  |  |  |
| KLT | 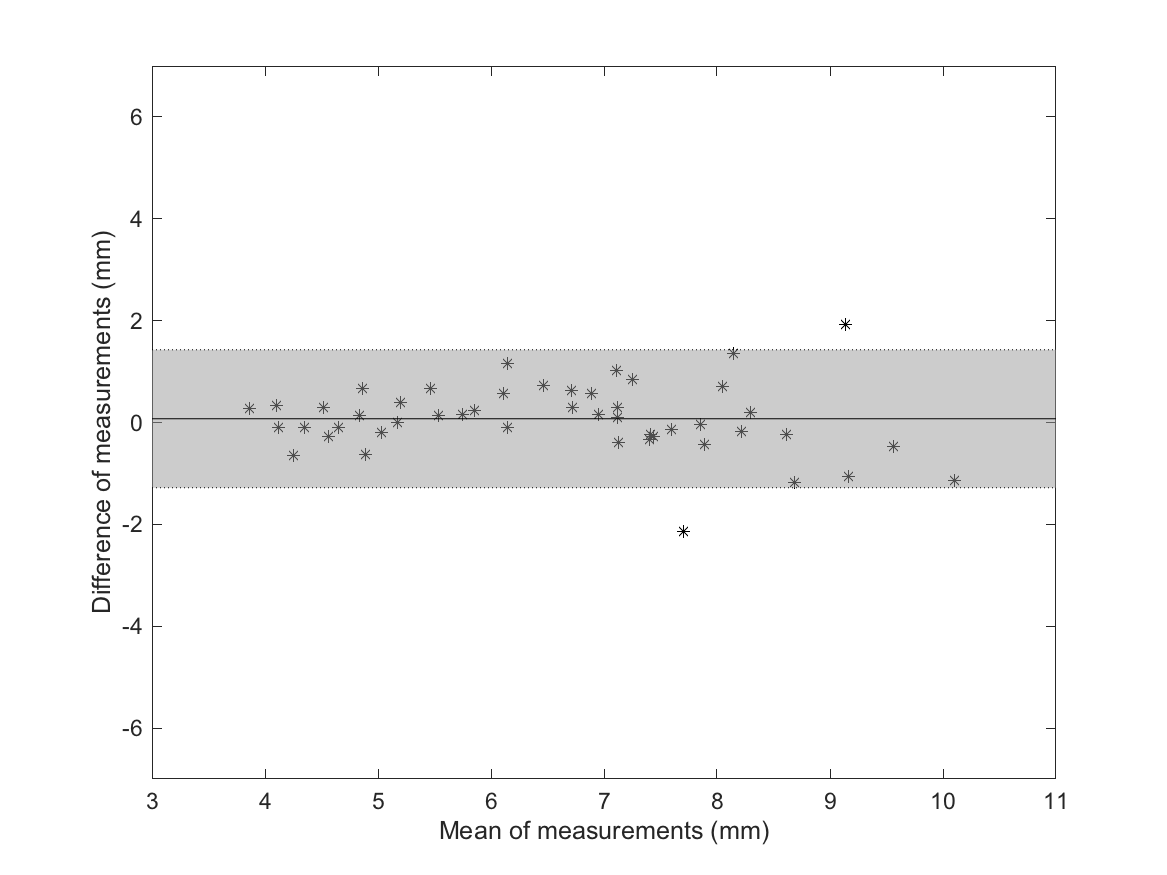 | 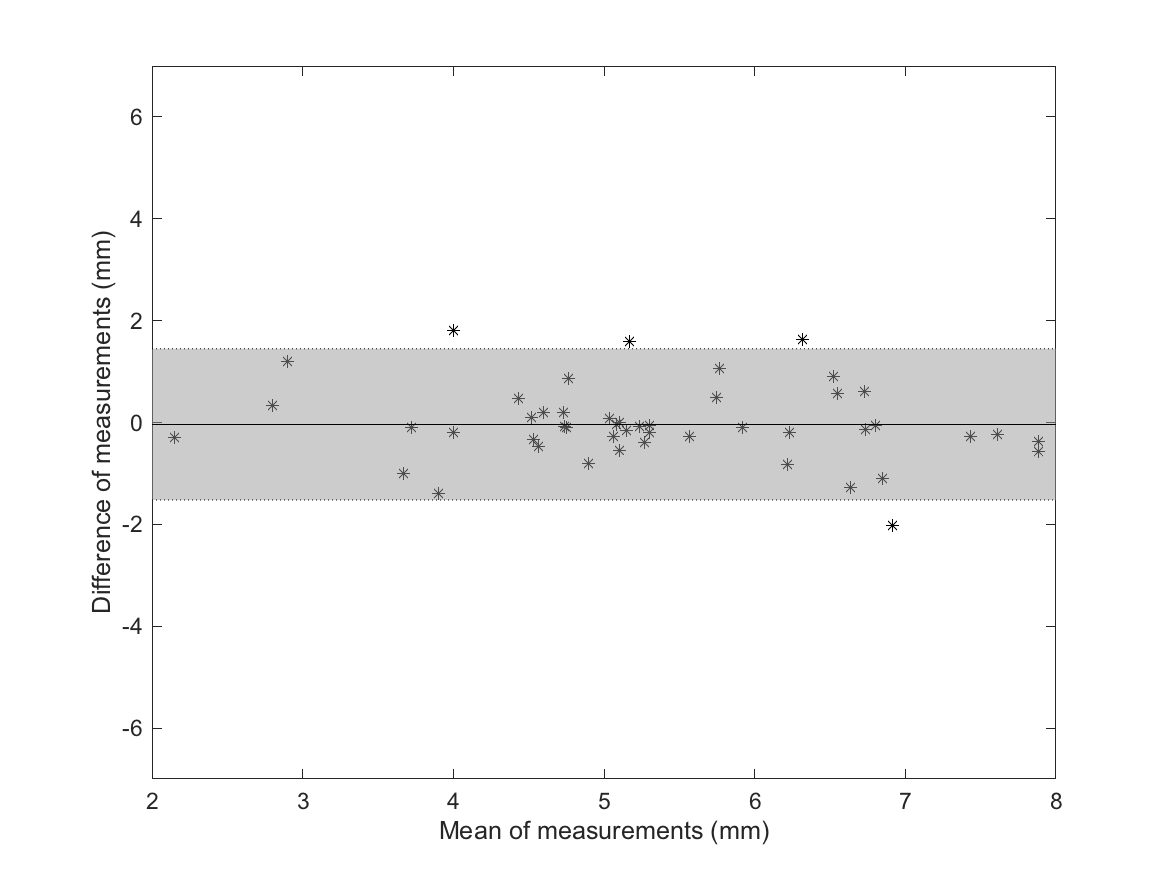 |
